# Phenome-wide association study (PheWAS) in EMR-linked pediatric cohorts, genetically links *PLCL1* to speech language development and *IL5-IL13* to Eosinophilic Esophagitis

**DOI:** 10.3389/fgene.2014.00401

**Published:** 2014-11-18

**Authors:** Bahram Namjou, Keith Marsolo, Robert J. Caroll, Joshua C. Denny, Marylyn D. Ritchie, Shefali S. Verma, Todd Lingren, Aleksey Porollo, Beth L. Cobb, Cassandra Perry, Leah C. Kottyan, Marc E. Rothenberg, Susan D. Thompson, Ingrid A. Holm, Isaac S. Kohane, John B. Harley

**Affiliations:** ^1^Center for Autoimmune Genomics and Etiology, Cincinnati Children's Hospital Medical CenterCincinnati, OH, USA; ^2^College of Medicine, University of CincinnatiCincinnati, OH, USA; ^3^Division of Biomedical Informatics, Cincinnati Children's Hospital Medical CenterCincinnati, OH, USA; ^4^Department of Biomedical Informatics, Vanderbilt University School of MedicineNashville, TN, USA; ^5^Department of Medicine, Vanderbilt University School of MedicineNashville, TN, USA; ^6^Center for Systems Genomics, The Pennsylvania State UniversityPhiladelphia, PA, USA; ^7^Division of Genetics and Genomics, Boston Children's HospitalBoston, MA, USA; ^8^Division of Allergy and Immunology, Department of Pediatrics, Cincinnati Children's Hospital Medical CenterCincinnati, OH, USA; ^9^Division of Genetics and Genomics, Department of Pediatrics, The Manton Center for Orphan Disease Research, Harvard Medical School, Boston Children's HospitalBoston, MA, USA; ^10^Children's Hospital Informatics Program, Center for Biomedical Informatics, Harvard Medical SchoolBoston, MA, USA; ^11^U.S. Department of Veterans Affairs Medical CenterCincinnati, OH, USA

**Keywords:** PheWAS, ICD-9 code, genetic polymorphism

## Abstract

**Objective:** We report the first pediatric specific Phenome-Wide Association Study (PheWAS) using electronic medical records (EMRs). Given the early success of PheWAS in adult populations, we investigated the feasibility of this approach in pediatric cohorts in which associations between a previously known genetic variant and a wide range of clinical or physiological traits were evaluated. Although computationally intensive, this approach has potential to reveal disease mechanistic relationships between a variant and a network of phenotypes.

**Method:** Data on 5049 samples of European ancestry were obtained from the EMRs of two large academic centers in five different genotyped cohorts. Recently, these samples have undergone whole genome imputation. After standard quality controls, removing missing data and outliers based on principal components analyses (PCA), 4268 samples were used for the PheWAS study. We scanned for associations between 2476 single-nucleotide polymorphisms (SNP) with available genotyping data from previously published GWAS studies and 539 EMR-derived phenotypes. The false discovery rate was calculated and, for any new PheWAS findings, a permutation approach (with up to 1,000,000 trials) was implemented.

**Results:** This PheWAS found a variety of common variants (MAF > 10%) with prior GWAS associations in our pediatric cohorts including Juvenile Rheumatoid Arthritis (JRA), Asthma, Autism and Pervasive Developmental Disorder (PDD) and Type 1 Diabetes with a false discovery rate < 0.05 and power of study above 80%. In addition, several new PheWAS findings were identified including a cluster of association near the *NDFIP1* gene for mental retardation (best SNP rs10057309, *p* = 4.33 × 10^−7^, *OR* = 1.70, 95%CI = 1.38 − 2.09); association near *PLCL1* gene for developmental delays and speech disorder [best SNP rs1595825, *p* = 1.13 × 10^−8^, *OR* = 0.65(0.57 − 0.76)]; a cluster of associations in the *IL5-IL13* region with Eosinophilic Esophagitis (EoE) [best at rs12653750, *p* = 3.03 × 10^−9^, *OR* = 1.73 95%CI = (1.44 − 2.07)], previously implicated in asthma, allergy, and eosinophilia; and association of variants in *GCKR* and *JAZF1* with allergic rhinitis in our pediatric cohorts [best SNP rs780093, *p* = 2.18 × 10^−5^, *OR* = 1.39, 95%CI = (1.19 − 1.61)], previously demonstrated in metabolic disease and diabetes in adults.

**Conclusion:** The PheWAS approach with re-mapping ICD-9 structured codes for our European-origin pediatric cohorts, as with the previous adult studies, finds many previously reported associations as well as presents the discovery of associations with potentially important clinical implications.

## Introduction

Phenome-wide association study (PheWAS) is a relatively new genomic approach to link clinical conditions with published variants (Denny et al., 2010). The concept, although not new, was originally applied to genomic research by the eMERGE (electronic MEdical Records and GEnomics) network, which is in a unique position to access tens of thousands of Electronic Medical Records (EMR) linked to ICD-9 codes in structured data. Multiple eMERGE PheWAS results have been published that primarily address adult cohorts (Denny et al., 2011, 2013). The phenotypic data used in PheWAS may include ICD-9 codes, epidemiologic data in health surveys, biomarkers, intermediate or quantitative traits (Pendergrass et al., 2011, 2013; Neuraz et al., 2013; Liao et al., 2014). By virtue of this inclusive approach, new hypotheses may be generated that provide insight into genetic architecture of complex traits. Challenges with PheWAS include multiple test corrections across the thousands of phenotypes tested and auto-correlation of some of the phenotypes. Nevertheless, novel robust insights have resulted from PheWAS, for example, genetic association findings with heart rate variability are notable (Ritchie et al., 2013).

PheWAS combines multiple phenotypes from previous GWAS, and identify common SNPs affecting different traits. In this study, we used this approach to evaluate whether known GWAS variants identified in adult diseases can be also identified in children using two EMR-linked pediatric datasets from eMERGE. PheWAS in pediatrics is particularly important because it not only assesses the effect of early age of onset on many established adult-GWAS loci, but also may provide insights into how a primary phenotype during child development develops into one or more diseases in adulthood. A priori, there are several reasons that in principle might make a pediatric PheWAS more challenging. These include the change in heritability with age for several traits (St Pourcain et al., 2014), the flux in the recommendations for pediatric monitoring for traits that are routinely measured in adults (Gidding, 1993; Klein et al., 2010) and the use of cross-sectional standardization rather than longitudinal standardization of developmental traits such as height (Tiisala and Kantero, 1971).

To determine whether robust association signals would be present in the context of these challenges, we conducted the first PheWAS study in pediatrics on our available samples. We successfully translated 93,724 specific ICD-9 diagnostic codes into 1402 distinct PheWAS code groups and 14 major disease concept paths and evaluated 2481 previously published variants. After quality control, only 2476 genetic variants were analyzed in 539 diseases in the two pediatric sites. Finally we replicated 24 genetic variants and identified 14 new possible associations confirming our hypothesis. Our primary results highlight the utility of an EMR-based PheWAS approach as a new line of investigation for discovery of genotype-phenotype associations in pediatrics.

## Materials and methods

### Study subjects

Protocols for this study were approved by the Institutional Review Boards (IRBs) at the institutions where participants were recruited. All study participants provided written consent prior to study enrolment; consent forms were obtained at each location under IRB guidelines. Children and teens, aged through 19 years old were included. The EMR-linked pediatric emerge cohorts consist of 4560 subjects from Cincinnati Children's Hospital Medical Center (CCHMC) and 1000 subjects from Boston Children's Hospital (BCH). Only those self-reported to have European ancestry were selected for this study (Table 1).

**Table 1:** **The demographic distribution of the European ancestry population (CCHMC-BCH)**.

	**Cohort names**	**#Europeans**	**M/F**	**Mean age (95%CI)**	**Array**
BCH^*^	The gene partnership	727	449/278	13.30(12.97–13.66)	Affymetrix-Axiom
CCHMC^**^	Cytogenetics	1228	758/470	7.32(7.03–7.62)	Illumina-610
	Cytogenetics	609	373/236	7.18(6.73–7.63)	Illumina-Omni-1
	EoE^†^	543	394/149	12.27 (11.70–12.67)	Illumina-Omni-5
	JIA^‡^	488	101/387	13.70(13.13–14.23)	Affymetrix-6
	Cincinnati- control cohorts	673	329/344	13.50(13.25–13.84)	Illumina-Omni-5
Total		4268	2403/1865	11.52(11.16–11.91)	

* BCH, Boston Children's Hospital;

** CCHMC, Cincinnati Children's Hospital Medical center;

† , Eosinophilic Esophagitis (EoE) cohorts;

‡ , Juvenile Idiopathic Arthritis cohorts (JIA).

The details of platforms used have been described elsewhere (Namjou et al., 2013).

### SNP prioritization

We limit our investigation to particular genetic variants: First, we obtained the list of all previously published SNPs from different public domain databases including The National Human Genome Research Institute (NHGRI) catalog of published Genome-Wide Association Studies (http://www.genome.gov/gwastudies), Genetic Association of Complex Diseases and Disorders (GAD, http://geneticassociationdb.nih.gov), the UCSC Genome Browser database (UCSC, http://genome.ucsc.edu/), Online Mendelian Inheritance in Man (OMIM, http://www.omim.org/), and PharmGKB (pharmgkb, https://www.pharmgkb.org). After linking this collection to PubMed reference numbers, only those with at least one reported of positive associations were selected regardless of the previously observed *p* values or number of publications. In addition, all downloaded databases were current at the time of this submission. From the filtered variants, 2476 variants were available and assessed in our clean, post-imputation genotyping dataset for analysis.

### Genotyping and statistical analyses

High throughput SNP genotyping was carried out previously in CCHMC and BCH using Illumina™ or Affymetrix™ platforms, as previously described (Namjou et al., 2013). Quality control (QC) of the data was performed before imputation. In each genotyped cohort, standard quality control criteria were met and single nucleotide polymorphisms (SNPs) were removed if (a) >5% of the genotyping data was missing, (b) out of Hardy-Weinberg equilibrium (HWE, *p* < 0.001) in controls, or a minor allele frequency (MAF) <1%. Samples with call rate <98% were excluded.

Recently all eMERGE cohorts have also undergone whole genome imputation. The details of these procedures are available in this issue of Frontiers in Genetics (Setia et al., 2014). Briefly, the imputation pipeline was implemented using IMPUTE2 program and the publicly available 1000-Genomes Project as the reference haplotype panel composed of 1092 samples (release version 2 from March 2012 of the 1000 Genomes Project Phase I, ftp://ftp-trace.ncbi.nih.gov/1000genomes/ftp/release/20110521) (Howie et al., 2011). The eMERGE imputed data provided to us were already filtered, i.e., imputed data with a threshold of 0.90 for the genotype posterior probability and with a IMPUTE2 info score > 0.7 (Howie et al., 2011). Principle component analysis (PCA) performed to identify outliers and hidden population structure using EIGENSTRAT (Price et al., 2006). The first two principle components explained most of the variance and were retained and used as covariates during the association analysis in order to adjust for population stratification. In addition, 14 outlier samples were removed. To illustrate the overall inflation rate a phenotype with sufficient number of cases and controls has been selected (autism) and the inflation of λ = 1.03 was obtained.

Next, from our prioritized SNP list mentioned above, 2481 variants were available. Five of these SNPs had a site-specific effect with either CCHMC or BCH (*p* < 10^−5^ for the difference between sites) and were removed from final analyses. For each phenotype, logistic regression was performed between cases and control adjusted for two principal components using PLINK (Purcell et al., 2007). To investigate whether either the phenotype or the genotype has an effect on the outcome variable, we perform phenotypic and genotypic conditional analyses, controlling for the effect of a specific SNP or phenotype. After pruning of highly correlated SNPs (*r*^2^ > 0.5), we used false discovery rate (FDR) methods to correct for multiple testing using the Benjamini–Hochberg procedure implemented in PLINK (Purcell et al., 2007). As a result of LD pruning 1828 independent variants were used for the purpose of FDR estimation. *Q* values correspond to the proportion of false positives among the results. Thus, *Q* values less than 0.05 signify less than 5% of false positives and are accepted as a measure of significance (FDR < 0.05) in this study. For any novel PheWAS findings, an adaptive permutation approach was performed using a sample randomization strategy in which case and control labels were permuted randomly (with up to 1,000,000 trials) in order to obtain empirical *p* values [PLINK (Purcell et al., 2007)]. We also report previous known effects that only produce suggestive findings in our study (0.05 < *p* < 0.001). Sample size and power calculations based on the size effect and risk allele frequency were estimated using QUANTO (Gauderman and Morrison, 2006). To graphically display results, LocusZoom was used (Pruim et al., 2010).

### Phenotyping

A phenome-wide association analysis (PheWAS) was performed in which presence or absence of each PheWAS code [mapped from translated ICD-9 codes as per Carroll et al., 2014)] were considered as a binary phenotype. The per-patient ICD-9 codes were obtained from the i2b2 Research Patient Data Warehouse at CCHMC and BCH. Also, these PheWAS codes were used to define comparison control groups by excluding the PheWAS case- code and those closely related to them in the ICD-9 hierarchy. Control groups for Crohn's Disease (CD), for instance, excluded CD, ulcerative colitis, and several other related gastrointestinal complaints. Similarly, control groups for myocardial infarction excluded patients with myocardial infarctions, as well as angina and other evidence of ischemic heart disease. The current PheWAS map and PheWAS script written in R is available [http://phewascatalog.org, (Carroll et al., 2014)]. In this study, subgroups of European cases with more than 20 samples were selected for PheWAS association study (539 subgroups) and the available published SNPs that passed quality controls were evaluated. The case cohorts for the two phenotypes of Juvenile Idiopathic Arthritis (JIA) and Eosinophilic Esophagitis (EoE) have both been previously published as parts of larger phenotype specific studies (Rothenberg et al., 2010; Thompson et al., 2012; Hinks et al., 2013). The origin of all case records is presented in Table 1. In this study, Juvenile Onset Rheumatoid Arthritis (JRA) is identified by ICD-9 codes and designated as JRA; when the criteria for Juvenile Idiopathic Arthritis (JIA) were applied in the studies of others (Thompson et al., 2012), then this phenotype was referred to as JIA.

### Results

**Figure 1:**
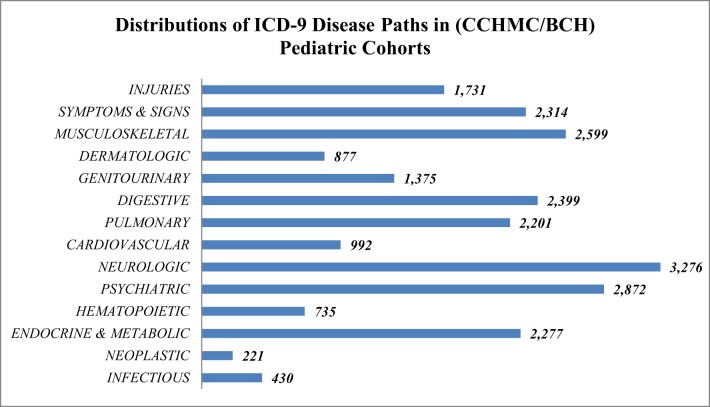
**Frequency and distribution of 14 major ontology concept path categories from CCHMC/BCH European pediatric cohorts**.

In this study only European ancestry was included in the analysis to avoid potential bias induced by ancestry. The demographic distribution of the European ancestry population under study (Table 2) had 93,724 specific ICD-9 diagnostic codes representing 1402 distinct PheWAS code groups and 14 major disease concept paths. The frequencies of concept path hierarchy of the ontology (Figure 1) show the neuropsychiatric concept path as the most frequent and neoplastic and infection paths as the least frequent.

**Table 2:** **Replication of previous GWAS association results in CCHMC/BCH pediatric cohorts**.

**Chr**	**SNP**	**Position**	**Gene**	**Minor allele**	**Case**	**Control**	***p* value**	**FDR *q* value**	**OR**	**Description**	**Case/ Control**
1	rs2476601	114377568	*PTPN22*	A	0.16	0.09	9.10E-07	8.01E-06	1.87 (1.46–2.41)	JRA	272 /3412
1	rs2476601	114377568	*PTPN22*	A	0.28	0.10	2.78E-05	4.16E-04	3.44 (1.80–6.57)	Thyroiditis	23/3571
1	rs2476601	114377568	*PTPN22*	A	0.18	0.10	0.007	NS	1.96 (1.16–3.31)	T1DM	47/3609
1	rs6679677	114303808	*PTPN22*	A	0.16	0.09	3.63E-07	4.15E-06	1.92 (1.49–2.47)	JRA	272/3412
1	rs6679677	114303808	*PTPN22*	A	0.28	0.10	2.00E-05	4.16E-04	3.52 (1.84–6.74)	Thyroiditis	23/3571
1	rs6679677	114303808	*PTPN22*	A	0.18	0.10	0.005	NS	2.00 (1.18–3.38)	T1DM	47/3609
2	rs3771180	102953617	*IL1RL1*	T	0.19	0.14	5.71E-05	0.0005	1.46 (1.19–1.80)	EoE or Food Allergy	599/2346
2	rs7574865	191964633	STAT4	T	0.32	0.24	0.004	NS	1.46 (1.11–1.92)	Wheezing	125/3372
3	rs78122814	85200034	*CADM2*	A	0.08	0.05	4.34E-05	0.0004	1.72 (1.32–2.24)	Autism	601/1840
5	rs3806932	110405675	*TSLP*	G	0.35	0.44	5.59E-07	8.38E-06	0.69 (0.59–0.80)	EoE	446/2586
5	rs272889	131665378	*SLC22A4*	A	0.46	0.37	1.53E-05	0.0003	1.45 (1.22–1.71)	Atopic Dermatitis	298/3031
5	rs12653750	131971902	*IL5-IL13*	T	0.27	0.20	9.74E-05	0.005	1.50 (1.22–1.84)	Eosinophilia	250/3344
6	rs75732170	101845494	*GRIK2*	A	0.06	0.03	8.49E-06	0.0002	2.00 (1.47–2.73)	Autism	601/1840
6	rs477515	32569691	*HLA-DRB1*	A	0.17	0.33	1.15E-12	8.62E-12	0.41 (0.32–0.53)	JRA	272/3412
6	rs477515	32569691	*HLA-DRB1*	A	0.07	0.33	1.12E-06	2.60E-05	0.16 (0.08–0.38)	Uveitis	51/3089
6	rs622137	32569852	*HLA-DRB1*	A	0.17	0.32	4.98E-13	5.78E-12	0.41 (0.32–0.53)	JRA	272/3412
6	rs2516051	32570184	*HLA-DRB1*	T	0.17	0.32	5.78E-13	5.78E-12	0.41 (0.32–0.53)	JRA	272/3412
6	rs2516049	32570400	*HLA-DRB1*	C	0.14	0.32	1.49E-15	4.48E-14	0.36 (0.27–0.46)	JRA	272/3412
6	rs660895	32577380	*HLA-DRB1*	G	0.42	0.21	7.85E-07	1.65E-05	2.73 (1.80–4.13)	T1DM	47/3609
6	rs9388489	126698719	*CENPW*	G	0.68	0.47	3.07E-05	0.0003	2.46 (1.58–3.80)	T1DM	47/3609
6	rs1490388	126835655	*CENPW*	T	0.68	0.47	4.29E-05	0.0003	2.42 (1.56–3.74)	T1DM	47/3609
9	rs7850258	100549013	*FOXE1*	A	0.15	0.34	0.005	NS	0.35 (0.15–0.78)	Thyroiditis	23/3571
9	rs1443438	100550028	*FOXE1*	T	0.15	0.34	0.009	NS	0.35 (0.15–0.78)	Thyroiditis	23/3571
10	rs12411988	65315397	*REEP3*	C	0.20	0.14	9.50E-05	0.005	1.53 (1.23–1.92)	JRA	272/3412
10	rs7903146	114758349	*TCF7L2*	T	0.44	0.29	0.001	NS	2.00 (1.29–3.08)	Abnormal Glucose Test	42/3609
16	rs12924729	11187783	*CLEC16A*	A	0.26	0.35	3.34E-08	9.08E-06	0.67 (0.58–0.77)	EoE or Food Allergy	599/2346
17	rs8067378	38051348	*GSDMB*	A	0.57	0.49	3.13E-06	0.0001	1.37 (1.19–1.57)	Asthma	499/3175
17	rs2290400	38066240	*GSDMB*	C	0.43	0.50	1.05E-05	0.0002	0.74 (0.64–0.84)	Asthma	499/3175
17	rs8074094	45348021	*ITGB3*	C	0.30	0.25	2.00E-05	0.0002	1.29 (1.15–1.45)	PDD	1141/1840
20	rs716316	14908741	*MACROD2*	T	0.32	0.39	2.01E-05	0.0003	0.74 (0.65–0.85)	Autism	601/1840

False discovery rate (FDR-q < 0.05) was set for the threshold of significance. The calculated odds ratio was based on minor allele frequency and the coded alleles were shown. All positions were based on NCBI build 37. NS (not significant). The p-values and q-values are ordered based on chromosome and position.

#### Replication of existing associations using PheWAS

We compared SNPs with previous GWAS-reports and present association findings (FDR-q < 0.05) after corrected for population stratification and standard quality control (Table 2).

First, for the two phenotypes of JRA and EoE samples overlap largely with those previously reported phenotype specific GWAS study (Rothenberg et al., 2010; Thompson et al., 2012; Kottyan et al., 2014). We reproduced the major findings of those publications using different methodology. For JRA, association with *PTPN22* is a consistent finding. As expected, we replicated a previous report of association of *PTPN22* at non-synonymous coding SNP rs2476601 with this phenotype and with the same direction of allele frequency, (*p* = 9.10 × 10^−7^, *OR* = 1.87, 95%CI 1.46 − 2.40). The SNP in proxy (rs6679677, *r*^2^ = 1) also produced a similar result (Table 2). In our cohorts, variants in *PTPN22* are also associated with thyroiditis as well as Type 1 diabetes mellitus (T1DM), consistent with previous reports and despite low sample size (Table 2) (Plenge et al., 2007; Todd et al., 2007; Lee et al., 2011). From these three known associations of *PTPN22*, i.e., JRA, T1DM, and thyroiditis, the largest magnitude of the association is with pediatric onset thyroiditis (Table 2, *OR* = 3.52 95%CI 1.84 − 6.75).

For JRA, multiple loci in the HLA region were also associated at the level of *p* < 10^−12^ including rs477515 and rs2516049 near *HLA-DRB1* (Table 2). Of note, the size effect of HLA related SNPs, were highest for those with coexisting uveitis (best SNP rs477515, *OR* = 6.5, 95% CI = 2.73 − 15.68 for the risk allele, Table 2). In addition, for JRA, another previously published association (rs12411988 in *REEP3*) was also found and with the same size effect as previously described (*OR* = 1.53) (Table 2) (Thompson et al., 2012).

Furthermore, with regard to EoE traits, we also replicated previous major finding of association of SNP rs3806932 located at the vicinity of the *TSLP* gene at 5q22 region [*p* = 5.59 × 10 − 7, *OR* = 0.69 (95%CI = 0.59 − 0.80)] in these cohorts (Table 2) (Rothenberg et al., 2010; Kottyan et al., 2014).

**Figure 2:**
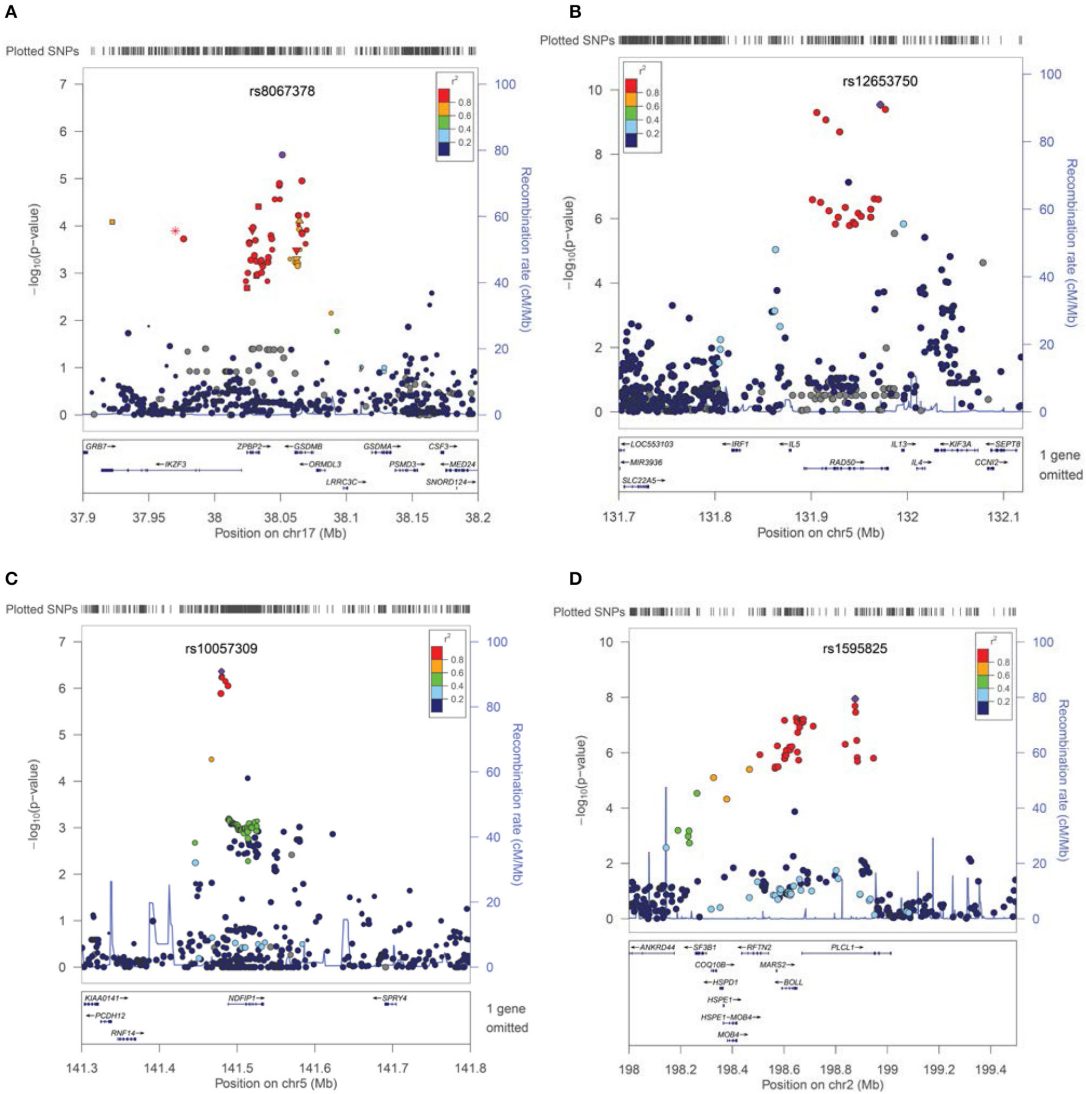
**Association results and signals contributing to Asthma, Eosinophilic Esophagitis, Mental Retardation, and Developmental Delays**. SNPs are plotted by position in a 0.2 Mb window against association signals (−log10 *P*-value). For each trait, the most significant SNP is highlighted. Estimated recombination rates (from HapMap) are plotted in cyan to reflect the local LD structure. The SNPs surrounding the most significant SNP, are color-coded to reflect their LD with identified SNP (taken from pairwise *r*^2^ values from the HapMap CEU database, www.hapmap.org). Regional plots were generated using LocusZoom (http://csg.sph.umich.edu/locuszoom). **(A**) Cluster of the association effect for asthma at 17q21 near the gasdermin-B (GSDMB) gene. **(B)** Association signal for Eosinophilic Esophagitis at 5q31 (IL5-IL13 cluster region). **(C)** Cluster of association near the NDFIP1 gene for Mental Retardation traits. **(D)** Plot of association effects in the PLCL1 region for Developmental Delays-Speech Disorders.

For asthma, the best PheWAS results were detected at 17q21 which includes *GSDMB* and has been previously reported to be associated specifically with childhood onset Asthma (Verlaan et al., 2009). In fact, the best associated SNP rs8067378 in our cohorts [*p* = 3.13 × 10^−6^, *OR* = 1.37 (1.19 − 1.57)], tags the asthma associated haplotype in which the allele-specific expression analyses for this haplotype has previously shown strong association with Asthma risk (Verlaan et al., 2009). There is strong support for this association from a cluster of variants in this neighborhood (Figure 2A).

The minor allele (T) of the intronic SNP rs7903146 in *TCF7L2* is one of the larger magnitude and more frequently identified associations in Type 2 diabetes mellitus (T2DM) and hyperlipidemia in many adult GWAS studies (Lyssenko et al., 2007; Huertas-Vazquez et al., 2008). In fact, the best PheWAS trait in our cohorts at this variant was also related to T2DM and hyperlipidemia as well, although our sample size was small. In this family of ICD-9 codes the best suggestive result was obtained for an abnormal glucose test with [*p* = 0.001, *OR* = 2.00 (95%CI 1.29 − 3.08)] (Table 2).

Specifically, for T1DM, in addition to the positive association with *PTPN22* mentioned above, additional published loci were confirmed and with relatively larger effect sizes (*OR* > 2) including known HLA-SNP rs660895 [*p* = 7.85 × 10^−7^, *OR* = 2.73 (95%CI = 1.80 − 4.13)], as well as variants near *CENPW* that previously have been reported for this trait (Table 2) (Barrett et al., 2009).

#### Other effects

Several loci previously associated with autism and pervasive developmental disorders (PDD) (GWAS or copy number variations reports) including those at *MACROD2, ITGB3, CADM2*, and *GRIK2* (Jamain et al., 2002; Weiss et al., 2006; Thomas et al., 2008; Anney et al., 2010) also provided evidence of association in our cohorts for these traits (Table 2). Variants in the *FOXE1* gene that have been previously associated with primary hypothyroidism and thyroiditis in adult eMERGE cohorts (Denny et al., 2011), produced a trend of association and consistent in directionality with thyroiditis in our pediatric cohorts despite low sample size (Table 2). No gene-gene interaction was evident between *PTPN22* and *FOXE1* for hypothyroidism in these data. Rs7574865 is a SNP in the third intron of the *STAT4* that has been associated with SLE and related autoimmune diseases (Namjou et al., 2009). In these cohorts, pediatric onset lupus was under-represented (less than 20 cases), however, suggestive associations with wheeze and asthma were detected [*p* = 0.004, OR 1.46 (95%CI = 1.11 − 1.92) (Table 2)] with the same direction of the difference in allele frequency previously observed in autoimmune traits. This possible association has also been reported in another study (Pykäläinen et al., 2005). Of note, in contrast to rheumatoid arthritis, the *STAT4* association effect was weak for JRA in our cohorts (effect size = 1.12, *p* = 0.17). GWAS studies have linked Inflammatory Bowel disease (IBD) to a number of IL-23 pathway genes, in particular *IL23R*. The well-known coding variant in the IL23 receptor (rs11209026) also showed a trend toward association with IBD in our cohorts with the same allelic direction but due to low sample size (31 cases) it did not reach significance (FDR-q > 0.05) (Li et al., 2010) (data not shown).

#### Novel findings from this PheWAS

A number of potentially novel associations remained significant after the permutation procedure to assess the probability of the observed distribution with beta > 0.8 FDR-q < 0.05 (Table 3). Variants in the Glucokinase Regulator gene (*GCKR*) have been previously implicated in metabolic disease, diabetes and hypertriglyceridemia in adults (Bi et al., 2010; Onuma et al., 2010) and were mostly associated with allergic rhinitis in our pediatric cohorts [best SNP rs780093 *p* = 2.18 × 10^−5^, *p*_(perm)_ = 8.06 × 10^−5^, *OR* = 1.39, 95%CI = (1.19 − 1.61)] (Table 3), while no significant association was found for diabetes. Indeed, conditional analyses, controlling for diabetes related traits suggest that this is an independent effect (*p*-conditional = 6.75 × 10^−5^). Another major regulatory locus for diabetes in adults, *JAZF1*, also was associated with allergic rhinitis in our cohorts (Table 3) even after controlling for diabetes (*p*-conditional = 8.46 × 10^−5^, for rs1635852). No significant gene-gene interaction was detected between these two loci or with *TCF7L2*.

**Table 3:** **Novel PheWAS findings in CCHMC/BCH pediatric cohorts**.

**Description**	**Case/Control**	**Chr**	**SNP**	**Position**	**Gene**	**Minor allele**	**Case**	**Control**	***p* value**	**p-permute**	Case needed^*^	**OR**
Allergic rhinitis	408/2754	2	rs1260326	27730940	*GCKR*	T	0.48	0.41	7.02E-05	1.21E-04	250	1.36 (1.17–1.58)
Allergic rhinitis	408/2754	2	rs780094	27741237	*GCKR*	T	0.47	0.40	2.94E-05	9.61E-05	250	1.38 (1.19–1.60)
Allergic rhinitis	408/2754	2	rs780093	27742603	*GCKR*	T	0.47	0.40	2.18E-05	8.06E-05	250	1.39 (1.19–1.61)
Allergic rhinitis	408/2754	7	rs864745	28180556	*JAZF1*	C	0.43	0.50	9.02E-05	1.11E-04	220	0.76 (0.65–0.88)
Allergic rhinitis	408/2754	7	rs1635852	28189411	*JAZF1*	C	0.43	0.50	6.58E-05	5.97E-05	220	0.75 (0.65–0.87)
Eosinophilic Esophagitis	446/2586	5	rs4143832	131862977	*IL5-IL13*	T	0.24	0.18	4.70E-06	1.70E-05	200	1.55 (1.29–1.87)
Eosinophilic Esophagitis	446/2586	5	rs12653750	131971902	*IL5-IL13*	T	0.28	0.19	3.03E-09	1.00E-06	100	1.73 (1.44–2.07)
Eosinophilic Esophagitis	446/2586	5	rs20541	131995964	*IL5-IL13*	A	0.26	0.19	3.72E-07	3.00E-06	150	1.61 (1.34–1.94)
Mental retardation	297/1840	5	rs11167764	141479065	*NDFIP1*	A	0.29	0.20	1.29E-06	4.00E-06	150	1.66 (1.35–2.04)
Mental retardation	297/1840	5	rs77110703	141479833	*NDFIP1*	T	0.29	0.20	5.83E-07	2.00E-06	150	1.69 (1.38–2.08)
Mental retardation	297/1840	5	rs10057309	141479870	*NDFIP1*	T	0.29	0.20	4.33E-07	2.00E-06	150	1.70 (1.39–2.09)
Developmental disorders	975/1840	2	rs1595825	198875464	*PLCL1*	A	0.15	0.21	1.13E-08	2.00E-06	150	0.65 (0.57–0.76)
Supporative otitis media	362/3082	1	rs10801047	191559356	*near RGS1*	A	0.13	0.08	1.61E-06	2.00E-06	250	1.77 (1.40–2.24)
Depression	107/2864	14	rs7141420	79899454	*NRXN3*	C	0.66	0.46	4.76E-05	1.10E-04	100	1.78 (1.34–2.34)

* P (permute): empirical permutation p values after case and control labels are permuted randomly (up to 1,000,000). All results were at the level of FDR-q < 0.05.

^**^“Cases needed” refers to the estimated number of cases needed to achieve 80% power to detect an association at alpha = 0.05 given the identified odds ratio and the MAF in this population.

Variants in a cytokine cluster of the *IL5-IL13* region, which is known to be associated with Asthma, Allergy, Atopic Dermatitis (AD) and Eosinophilia, produced a cluster of association with EoE in our cohorts [best SNP rs12653750, *p* = 3.03 × 10^−9^, *p*_(perm)_ = 1.00 × 10^−6^, *OR* = 1.73 (1.44 − 2.07)] (Bottema et al., 2008; Granada et al., 2012). There is a cluster of significant variants in this neighborhood of chromosome 5 (5q31) associated with EoE (Figure 2B). In our cohorts, weaker associations can be detected for all allergy-related phenotypes with the association with Eosinophilia being the most impressive [*p* = 9.74 × 10^−5^ (Table 2)]. However, conditional analyses and controlling for Asthma and Eosinophilia suggest that an independent effect still exists for EoE at this locus using EMR data (conditional *p* = 9.74 × 10 - 5 for rs20541). Moreover, no long distance linkage disequilibrium between rs3806932 in *TSLP* gene at 5q22 and rs20541 was detected in this population (*r*^2^ = 0.0002, *D*' = 0.02).

We also observed association with AD within this cytokine cluster consistent with previous reports (Paternoster et al., 2011). However, the best associated SNP for AD (rs272889) was located at *SLC22A4* in our population (Table 2). These two variants, rs272889 and rs12653750, were separated by more than 300kb with low linkage disequilibrium (*r*^2^ < 0.1). A residual effect still exists for AD and rs272889 after controlling for EoE status or the rs12653750 variant that suggests a distinct effect (*p*-conditional = 0.002). Noteworthy, with regard to AD, another reported SNP (rs2897442) downstream of this cluster at *KIF3A* gene produced only a suggestive association (*p* = 0.005) in our cohort (data not shown).

Because of the pleotropic effects between EoE and other allergy related traits, in addition to conditional analyses, we also found possible synergistic effects. One of the closely related phenotypes with EoE is the presence of food allergy. When we combined these two as a subgroup, two additional effects were identified. One cluster was in *IL1RL1* that was previously associated with the related phenotype, i.e., allergy and asthma (best SNP rs3771180, *p* = 5.71 × 10^−5^, Table 2, Torgerson et al., 2011) and another was in *CLEC16A*, previously associated with different autoimmune diseases [best SNP rs12924729, *p* = 3.34 × 10^−8^ (Table 2), (Mells et al., 2011)] and was reported as a suggestive effect in recent GWAS study for EoE (Kottyan et al., 2014).

Variants near *RGS* cluster of genes on chromosome 1, previously reported to be associated with IBD and other autoimmune diseases (Hunt et al., 2008; Esposito et al., 2010), were associated with susceptibility to infection, in particular suppurative otitis media [best SNP rs10801047, *p* = 1.61 × 10^−6^, *p*_(perm)_ = 2.00 × 10^−6^, *OR* = 1.77 95%CI = 1.398 − 2.24].

New association signals have been detected near the *NDFIP1* gene for mental retardation related traits. Variants near this gene that is expressed mostly in brain, were previously reported to be associated with IBD through an unknown mechanism and with a risk effect for major allele (SNP = rs11167764) (Franke et al., 2010). Instead, we found a risk effect for the minor allele [best SNP rs10057309, *p* = 4.33 × 10^−7^, *p*_(perm)_ = 2.00 × 10^−6^, *OR* = 1.702, 95%CI = 1.38 − 2.09] (Table 3). Similarly, cerebral palsy, which is linked to mental retardation, was also associated with this variant (*p* = 9.00 × 10^−4^). However, conditional analyses controlling for cerebral palsy suggest an independent effect for overall mental retardation (conditional *p* = 8.00 × 10^−4^). Furthermore, excluding the small number of samples with known chromosomal abnormalities (*N* < 40) did not affect this result. The overall cluster effect in this neighborhood for mental retardation bolsters the suspicion that an association is found here (Figure 2C).

Additionally, for developmental delays of speech and language, a novel signal effect was detected in the *PLCL1* gene at chromosome 2 [best SNP rs1595825, *p* = 1.13 × 10^−8^, *OR* = 0.65 (0.57 − 0.76)] (Figure 2D, Table 3). Weaker associations (0.01 > *p* > 0.00001) were also detected for related neurologic phenotypes including abnormal movement, lack of coordination and epilepsy at this locus (data not shown).

*NRXN3* polymorphisms that have been previously reported to be associated with substance dependence (Docampo et al., 2012), smoking behavior and attention related problems (Stoltenberg et al., 2011), were associated with depression in our pediatric cohorts (Table 3 Noteworthy, the major allele of our reported SNP (rs7141420) has been linked to obesity in adult cohorts (Berndt et al., 2013), while we found association with the minor allele for depression [*p* = 4.76 × 10^−5^, *OR* = 1.78 (1.34 − 2.34), Table 3]. Furthermore, rare micro-deletions in this gene were previously reported for Autism case reports but these rare variants are not available to assess in our genotyped cohorts (Vaags et al., 2012).

## Discussion

This first pediatric PheWAS finds 38 associations, 24 previously known phenotype-genotype associations in a pediatric population using EMR-linked eMERGE databases and identified 14 new possible associations at beta > 0.8 and FDR-q < 0.05. From analysis performed on EMR-linked data from 4268 European individuals, we successfully confirmed several major effects for phenotypes with moderate to large sample size, in particular for Asthma, Autism, and neurodevelopmental disease as well as several effects for Type 1 and Type 2 Diabetes (T1DM, T2DM) and Thyroiditis. Almost all of the significant phenotype associations were with common variants (MAF > 10%) (Tables 2, 3). In addition, we compared and verified the consistency of allele frequency of reported markers among cohorts, sample collection sites and with CEU-Hapmap data. Considering a desired power of 0.8, for variants at the fixed allele frequency of 10% and size effect of 1.5 or above, 200 cases are sufficient to detect association at an alpha level of 0.05. Indeed, we have surpassed this level for most of our reported traits. In addition, for all reported phenotypes the control sample was at least two or three times larger than cases (Tables 2, 3). Importantly, since our control samples for each trait are an EMR-derived population and not healthy individuals, this large number of control samples provides minor allele frequencies consistent with hapmap-CEU frequencies for all of our reported variants.

The results for JRA and EoE depend upon previously published studies of these phenotypes. While the case samples are mostly identical, the control samples were substantially different. Consequently, we cannot refer to these particular findings as constituting confirmation and yet our results and different methodology support the previous reports.

In addition, we also identified several novel PheWAS findings for pediatric traits in particular for Allergic Rhinitis, Otitis Media, EoE, Mental Retardation, and Developmental Delays all with sufficient power (beta > 0.8) (Table 3, Figures 2B–D). This study, however, is underpowered to make discoveries for rare variants or uncommon traits. The power to detect a finding in PheWAS is determined by many factors, including sample size, risk allele frequency, effect size, model of inheritance, the effect of environment and the prevalence of a phenotype within the population.

Similar to previous studies, we also observed pleiotropy for a number of loci in particular *PTPN22* for JRA, T1DM, and Thyroiditis, IL5 for Eosinophilia, Asthma, and EoE and *NDFIP1* for Mental Retardation traits and Cerebral Palsy. These pleotropic effects are specifically expected to be due to underlying biologic correlations. On the other hand, we rarely observed simultaneous robust associations with multiple unrelated phenotypes that had sufficient power. Furthermore, one of the advantages of PheWAS studies is the ability to control the granularity of a database with regard to related phenotypes. For example, by combining two related phenotypes such as uveitis with JRA or food allergy with EoE, we were able to evaluate new subgroups and identify new loci responsible for shared underlying pathways that otherwise cannot be detected or require much larger sample sizes. Further studies with larger sample sizes would be useful to test and perhaps corroborate these findings.

Association of Allergic Rhinitis with loci responsible for diabetes in adults (*GCKR-JAZF1*) may highlight a shared underlying mechanism. In fact, the connection between allergy and diabetes has been previously suggested in humans but cannot be explained by the Th1/Th2 paradigm (Dales et al., 2005). Moreover, in animal experiments, treating mice with mast cell-stabilizing agents reduced diabetes manifestations (Liu et al., 2009). It is also possible that in our pediatric cohorts we have under-diagnosed children who are diagnosed with diabetes which would appear in a later stage of development. In fact, *GCKR* is an inhibitor of glucokinase (*GCK*), a gene responsible for the autosomal dominant form of T2DM that usually develops later in life and in adulthood. Of note, neither of these two loci showed significant association with Body Mass Index (BMI) in our previous report with these data nor has the obesity link been established in adult studies (Namjou et al., 2013).

The novel association of a cytokine cluster in the *IL5-IL13* region for the EoE trait is particularly interesting since anti-IL5 monoclonal antibodies have been recommended as a novel therapeutic agent for EoE and other eosinophilia–related traits (Corren, 2012). In general, both *IL5* and *IL13* play a major role for regulation of maturation, recruitment, and survival of eosinophils and the variant reported here has been previously associated with other allergic-related traits and with the same direction of allele frequency difference (Bottema et al., 2008; Granada et al., 2012). In particular, a non-synonymous polymorphism in the IL13 gene, rs20541 (R130Q) (Table 3), has been shown to be associated with increased IL-13 protein activity, altered IL-13 production, and increased binding of nuclear proteins to this region (van der Pouw Kraan et al., 1999). Perhaps, the association is a reflection of linkage disequilibrium with another polymorphism in the 5q31 region. In fact, in our analyses residual effect still exists for the best SNP (rs12653750), shown in Figure 2B after controlling for rs20541 (*p*-conditional = 2.27 × 10^−5^) (*r*^2^ = 0.35). This possible association did not reach significance in previous GWAS studies for EoE and had only produced a suggestive effect (0.05 < *p* < 0.001). Perhaps, this behavior is explained partly by phenotypic heterogeneity since minor allele frequency of independent set of both control populations were the same. Indeed, we found that those with the subphenotype of EoE with Eosinophilia had the strongest size effect (*OR* = 1.83, 95%CI = 1.44 − 2.32) and our cohorts were enriched with this subphenotype [177 of total 446 EoE cases (40%)]. Of note, the SNPs in this region were originally selected because of eosinophilia-related publications (Bottema et al., 2008; Granada et al., 2012).

Moreover, combining subgroups of patients with food allergy and EoE revealed two new loci that may explain shared etiology. Indeed, the connection between allergy and Interleukin 1 receptor-like-1 (*IL1R1*) is already known (Torgerson et al., 2011). The ligand for IL1R1, IL-33, is a potent eosinophil activator (Bouffi et al., 2013). Interestingly, there is also a report of association of *CLEC16A* variants with allergy in large analysis with more than 50,000 subjects from 23andMe Inc. (Hinds et al., 2013). C-type lectin domain family 16, also known as *CLEC16A*, is mostly associated with autoimmune related traits and is highly expressed in B lymphocytes and natural killer cells. The molecular and cellular functions of CLEC16A are currently under investigation.

Our conditional analyses suggest an independent effect at the *SLC22A4* gene for Atopic Dermatitis. This solute carrier family gene is predominantly expressed in CD14 cells and has an important role for elimination of many endogenous small organic cations as well as a wide array of drugs and environmental toxins. The associated SNP, rs272889, has been previously shown to be correlated with blood metabolite concentration (Suhre et al., 2011). Other variants in this gene were associated with IBD and Crohns disease as well (Feng et al., 2009). Of note, a key substrate of this transporter is ergothioneine, a natural antioxidant, which Mammalia acquire exclusively from their food. Ergothionine is a powerful antioxidant though its precise physiological purpose remains unclear.

Asthma is associated at the 17q21 in our cohorts (Figure 1). The best associated SNP, rs8067378, is known to function as a cis-regulatory variant that correlates with expression of the *GSDMB* gene (Verlaan et al., 2009). Variants in *GSDMB* have been shown to determine multiple asthma related phenotypes specifically in childhood asthma including associations with lung function and disease severity (Tulah et al., 2013). These gasdermin-family genes are implicated in the regulation of apoptosis mostly in epithelial cells and have also been linked to cancer; however, their actual function with respect to disease association remains unknown. The associated variants in this cluster are suspected to be regulatory SNPs that govern the transcriptional activity of at least three nearby genes (*ZPBP2, GSDMB, and ORMDL3*) (Verlaan et al., 2009).

We confirmed several loci responsible for Autism and Pervasive Developmental Disease including *MACROD2, ITGB3, CADM2, and GRIK2. ITGB3* has been known as a quantitative trait locus (QTL) for whole blood serotonin levels (Weiss et al., 2004, 2006). Serotonin is a monoamine neurotransmitter that has long been implicated in the etiology of Autism. In fact, about 30 percent of patients with autism have abnormal blood serotonin levels (Weiss et al., 2004). Similarly, *GRIK2* is an ionotropic glutamate receptor associated with autism (Cook, 1990; Cook et al., 1997). *CADM2* is a member of the synaptic cell adhesion molecule with roles in early postnatal development of the central nervous system (Thomas et al., 2008). The function of *MACROD2* (previously c20orf133) is still largely unknown. For Autism that is more commonly seen in males, we found no significant gender effect for these loci.

Association of variants in the neighborhood of RGS cluster genes with suppurative otitis media is another novel finding. SNPs in this region have been previously linked to celiac disease, multiple sclerosis and other autoimmune diseases (Hunt et al., 2008; Esposito et al., 2010). The link between susceptibility to infection and autoimmunity has been long suggested given the fact that the level and regulation of RGS proteins in lymphocytes also significantly impact lymphocyte migration and function. In our pediatric cohort the number of patients with celiac disease was small (*n* = 23) and the association was not detected. Interestingly, one of the major risk variant for celiac disease, rs13151961 (KIAA1109), as well as known HLA variants, produced a tread toward association for celiac disease but did not pass the FDR threshold (data not shown).

Finally we also detected a novel association between mental retardation and the *NDFIP1* gene (Figure 2C, Table 3). Of note, no effect was detected with Autism at this locus. Indeed, the only other effect observed in this region was related to Cerebral Palsy (*p* = 9.00 × 10^−4^) and, as mentioned above, an independent effect exists for Mental Retardation. The PheWAS code for mental retardation includes ICD-9 codes for mild, moderate and profound degrees of retardation as well as not-otherwise-specified (MR-NOS). Indeed, an additive correlation can also be detected when we score these subgroups according to severity excluding the MR-NOS subgroup (*p* = 3.00 × 10^−4^). Larger sample size is necessary to fully elucidate this interesting effect. The Nedd4 family-interacting protein 1 (Ndfip1) is an adaptor protein for the Nedd4 family of E3 ubiquitin ligases important for axon and dendrite development. In fact, cerebral atrophy is one of the main findings in Ndfip1 KO mice (Hammond et al., 2014). Another neurodevelopmental association effect was observed in the vicinity of the Phospholipase C-Like 1 (PLCL1, PRIP-1) gene for overall Developmental Delays-Speech and Language Disorder (Table 3, Figure 2D). This gene which is expressed predominantly in brain, regulates the turnover of GABA-receptors, contributes to the maintenance of GABA-mediated synaptic inhibition, and has been implicated in several pathologies in animal models and human including epilepsy, bone density and cancer (Liu et al., 2008; Zhu et al., 2012). Finally, we also detected a link between Neuroxin-3 and early onset depression in this study (Table 3). In fact, this gene has a major role in synaptic plasticity and function in the nervous system as a receptor and cell adhesion molecule.

In summary, by using the PheWAS approach and re-mapping the ICD-9 codes on our European ancestry pediatric cohorts we have been able to verify and confirm a variety of previously reported associations as well as discover new effects that potentially have clinical implications. Similar to adult PheWAS studies, our data also support the importance of this approach in pediatrics. We replicated known phenotype-genotype associations in a pediatric population using these EMR-linked eMERGE databases, and also noted a number of new possible associations that warrant additional study, especially including the relationship of *PLCL1* to speech and language development and *IL5-IL13* to EoE. Some of the limitations to the current PheWAS map include the fact that current map does not take into account of the correlation between some phenotypes and treat them as independent. Future pediatric PheWAS directions will include enhancements of a PheWAS map for more precise modeling of trait associations as well as improvements for richer querying and filtering.

### Conflict of interest statement

The Guest Associate Editor Mariza De Andrade declares that, despite having collaborated with authors Bahram Namjou, Joshua C. Denny, Leah C. Kottyan, Marylyn D. Ritchie, and Shefali S. Verma, the review process was handled objectively and no conflict of interest exists. The Review Editor Andrew Skol declares that, despite having collaborated with author John B. Harley, the review process was handled objectively and no conflict of interest exists. Marc E. Rothenberg is a consultant for Immune Pharmaceuticals and has an equity interest. Marc E. Rothenberg has a royalty interest in reslizumab being developed by Teva Pharmaceuticals. Marc E. Rothenberg, John B. Harley, and Leah C. Kottyan are co-inventors of a patent application, being submitted by CCHMC, concerning the genetics of EoE. The authors declare that the research was conducted in the absence of any commercial or financial relationships that could be construed as a potential conflict of interest.
